# 

**Published:** 1880-01

**Authors:** 


### BOOKS AND PAMPHLETS RECEIVED.

Transactions of Illinois State Medical Society, May 20 and 21, 1879.

Infant Feeding and its Influence on Life, or the Causes and Prevention of Infant Mortality. By C. H. T. Routh, M.D. Third edition; 1879. Cloth, pp. 270. New York: Wm. Wood & Co. Chicago: W. T. Keener.

The Physician's Visiting List for 1880. Twenty-ninth year of its publication. Philadelphia: Lindsay & Blakiston.

Treatise on the Theory and Practice of Medicine. By John Syer Bristowe, M.D., London. Second American edition, revised by the author, with notes and additions by J. H. Hutchinson, M.D. 1879. Leather, pp. 1081 Philadelphia: H. C. Lea.

Transactions of the Medical Society of the State of Pennsylvania, at its Thirteenth Annual Session, May, 1879. Vol. XII, part II. Published by the Society.

Dr. Houts' Physician's Journal, Credit Book and Ledger. Third improved edition.

New and Improved Surgical Instruments. By J. G. Meachem, sr., M.D. Reprint from Transactions of Wisconsin State Medical Society.

Report of the Adams County Medical Society. By J. W. C. O'Neal, M.D. Reprint from the Transactions of the Medical Society of the State of Pennsylvania for 1879.

A Biographical Dictionary of Contemporary American Physicians and Surgeons. Edited by Wm. B. Atkinson, M.D. Second edition, enlarged and revised. Cloth, pp. 767. Philadelphia: D. G. Brinton. Chicago: Jansen, McClurg & Co. Price, $5.00 cloth; $6.00 leather.

Dictionary of German Terms used in Medicine. By Geo. R. Cutter, M.D. $3.00, cloth; 1879. New York: G. P. Putnam & Sons. Chicago: Jansen, McClurg & Co.

A Text Book of Physiology. By M. Foster, M.A., M.D. With illustrations. Third edition, revised. Cloth, pp. 720, price $3.50; sheep, $6.50. Cheaper students' edition to follow. London: McMillan & Co. Chicago: Jansen, McClurg & Co.

---

A NEW HEALTH RESORT.—The London *Medical Press and Circular* for Oct. 29, 1879, publishes as the title of one of its leaders, "Residence Abroad in Lung Consolidation!" We should prefer a residence at home in the "solid South."

### ANNOUNCEMENTS FOR THE MONTH.

### SOCIETY MEETINGS.

Chicago Medical Society—Mondays, Jan. 5 and 19.
West Chicago Medical Society—Mondays, Jan. 12 and 26.

### CLINICS.

MONDAY.

Eye and Ear Infirmary—2 p. m., Ophthalmological, by Prof. Holmes; 3 p. m., Otological, by Prof. Jones.
Mercy Hospital—2 p. m., Surgical, by Prof. Andrews.
Rush Medical College—2 p. m., Dermatological and Venereal, by Prof. Hyde; 3 p. m., Medical, by Dr. Bridge.
Woman's Medical College—3 p. m., Diseases of the Chest, Prof. Ingals.

TUESDAY.

Cook County Hospital—2 to 4 p. m., Medical and Surgical Clinics.
Mercy Hospital—2 p. m., Medical, by Prof. Hollister.

WEDNESDAY.

Chicago Medical College—2 p. m., Eye and Ear, by Prof. Jones.
Rush Medical College—3:30 to 4:30 p. m., Diseases of the Chest, by Dr. E. Fletcher Ingals.

THURSDAY.

Chicago Medical College—2 p. m., Gynæcological, by Prof. Jenks.
Rush Medical College—3 p. m., Diseases of the Nervous System, by Prof. Lyman.
Eye and Ear Infirmary—2 p. m., Ophthalmological, by Dr. Hotz.
Woman's Medical College—3 p. m., Surgical, by Prof. Owens.

FRIDAY.

Cook County Hospital—2 to 4 p. m., Medical and Surgical Clinics.
Mercy Hospital—2 p. m., Medical, by Prof. Davis.

SATURDAY.

Rush Medical College—2 p. m., Surgical, by Prof. Gunn.
Chicago Medical College—2 p. m., Surgical, by Prof. Isham; 3 p. m., Neurological, by Prof. Jewell.
Woman's Medical College—11 a. m., Ophthalmological, by Prof. Montgomery; 2 p. m., Gynæcological, by Prof. Fitch.

Daily Clinics, from 2 to 4 p. m., at the Central Free Dispensary, and at the South Side Dispensary.

